# Analysis of the expression and prognostic value of MT1-MMP, β1-integrin and YAP1 in glioma

**DOI:** 10.1515/med-2022-0449

**Published:** 2022-03-09

**Authors:** Yangyang Zhai, Wei Sang, Liping Su, Yusheng Shen, Yanran Hu, Wei Zhang

**Affiliations:** Department of Pathology, First Affiliated Hospital, Xinjiang Medical University, Urumqi, Xinjiang, 830054, P. R. China; State Key Laboratory of Etiology and Prevention of High Incidence in Central Asia, Xinjiang Medical University, 830000, P. R. China; Department of Neurosurgery, First Affiliated Hospital, Xinjiang Medical University, Urumqi, Xinjiang 830054, P. R. China; Xinjiang Medical University, Urumqi, The Xinjiang Uygur Autonomous Region of China, 830011, P. R. China

**Keywords:** glioma, immunohistochemistry, prognosis, molecular characteristics, *MT1-MMP*, *YAP1*, *β1-integrin*

## Abstract

Increased expression of membrane type 1-matrix metalloproteinase (*MT1-MMP*/*MMP14*) is associated with the development of many cancers. *MT1-MMP* may promote the entry of yes-associated protein1 (*YAP1*) into the nucleus by regulating the regulation of *β1-integrin*. The purpose of this study was to investigate the effects of *MT1-MMP*, *β1-integrin* and *YAP1* on the prognosis of gliomas. The expression of proteins was detected by bioinformatics and immunohistochemistry. The relationship between three proteins and clinicopathological parameters was analyzed by the *χ* ^2^ test. Survival analysis was used to investigate the effects of three proteins on prognosis. The results showed that high expressions of *MT1-MMP*, *β1-integrin* and *YAP1* were found in glioblastoma (GBM) compared with lower-grade glioma (LGG). There was a significantly positive correlation between *MT1-MMP* and *β1-integrin* (*r* = 0.387), *MT1-MMP* and *YAP1* (*r* = 0.443), *β1-integrin* and *YAP1* (*r* = 0.348). Survival analysis showed that patients with overexpression of *MT1-MMP*, *β1-integrin* and *YAP1* had a worse prognosis. *YAP1* expression was the independent prognostic factor for progression-free survival (PFS). There was a statistical correlation between the expression of *MT1-MMP* and *YAP1* and isocitrate dehydrogenase 1 (*IDHl*) mutation. Thus, this study suggested that *MT1-MMP*, *β1-integrin* and *YAP1*, as tumor suppressors, are expected to be promising prognostic biomarkers and therapeutic targets for glioma patients.

## 1 Introduction

Glioma is the most common primary intracranial tumor. Glioma is classified into four grades (I to IV) by the World of Health Organization (WHO) [1]. Glioblastoma, which belongs to WHO IV, is the most aggressive glioma. It blurs the boundary between the tumor tissue and the surrounding normal brain, which makes it difficult to treat with all existing treatments, and the median survival time for patients with this kind of tumor is 12–15 months [2]. Different from GBM, WHO II grade and WHO III grade have a better prognosis, with an average survival of 78.1 and 37.6 months, respectively, in terms of treatment [3]. Therefore, grades II and III are considered to be lower-grade gliomas (LGG) [4,5,6,7]. More and more studies have proved that the molecular typing of glioma plays an irreplaceable role in its development, such as *IDH1* [8].

The development and malignant progression of gliomas are related to its secretion of a variety of matrix metalloproteinases (MMPs) involving the degradation of extracellular matrix (ECM) components [9,10,11]. *MT1-MMP*, one of the most typical members of thin-film matrix metalloproteinases, not only reduces a variety of ECM components but also activates matrix metalloproteinase-2 (*MMP-2*). In addition, *MT1-MMP* plays an important role in the development and invasion of these cancers such as colorectal cancer, pancreatic cancer, breast cancer and oral squamous cell carcinoma [12,13,14,15]. Previous studies have shown that *MT1-MMP* is one of the most important MMP in promoting the occurrence and development of many kinds of tumors, and its expression is closely related to poor prognosis and tumor metastasis [16,17,18,19]. Recent studies have also shown that there is a direct correlation between *MT1-MMP* and *β1-integrin*. *MT1-MMP* accumulates at the bud tip of the invasive mammary gland, thus activating *β1-integrin* to promote cell invasive sprouting to coordinate interstitial invasion [20].

*YAP1* is a key effector downstream of the Hippo signaling pathway, which is often activated during the growth and development of many solid tumors. It is also an important driving factor to increase the proliferation and invasion of tumor cells and tumor drug resistance [21,22,23]. The regulation of the Hippo signaling pathway is known to be mediated by phosphorylation and subcellular localization of *YAP1*. Activation of the Hippo signaling pathway induces phosphorylation of *YAP1*, which prevents translocation to the nucleus. When the Hippo signal pathway is inactivated, dephosphorylated *YAP1* translocates to the nucleus and interacts with transcription factors, resulting in cell proliferation in various organ systems [24]. It has been found that the increased expression of *YAP1* can promote the proliferation and migration of bladder cancer cells [25]. Numerous studies have shown that upregulation of *YAP1* can induce epithelial–mesenchymal transformation (EMT), inhibit apoptosis and promote the production of tumor stem cells [26,27,28]. These concepts inform possible strategies for effectively inhibiting *YAP1* activity in cancer patients, such as monotherapy and combination therapy of conventional chemotherapy, radiotherapy or immunotherapy. However, there are a few reports on the expression of *YAP1* in gliomas and its correlation with *MT1-MMP*.

Recent reports have provided evidence of a link between the Hippo/YAP1 signaling pathway and *MT1-MMP*. *YAP1* and *MT1-MMP* could cooperate to promote tumor cells invasion and metastasis. By effecting ECM remodeling, *MT1-MMP* regulates stem cell shape, thereby activating a *β1-integrin/Rho GTPase* signaling cascade and triggering the nuclear localization of the transcriptional coactivators *YAP1* and *TAZ*. Both *in vitro* and *in vivo*, *MT1-MMP* as an upstream and necessary activating cofactor, loss of *MT1-MMP* leads to a significant decrease in the level of *YAP/TAZ* [29]. *β1-integrin* (*ITGB1*) is a transmembrane glycoprotein that induces the phosphorylation of focal adhesion kinase and *AKT* in cell growth and apoptosis [30]. *β1-Integrin* has a variety of functions in cell adhesion, contact and anchorage-dependent cell survival, and regulates various cellular processes including invasion, metastasis and angiogenesis. Increasing evidence suggests that *β1-integrin* plays a key role in the development of cancer. The increased expression of *β1-integrin* in a variety of human cancers, such as prostate cancer and cutaneous malignant melanoma, can promote the malignant progression and metastasis of tumors. It has also been shown that high *β1-integrin* expression is significantly associated with worse OS in lung cancer and breast cancer [31]. However, the related mechanism underlying *MT1-MMP*-and *β1-integrin*-mediated development of GBM is unclear.

In this study, we investigated the expression of MMPs in the TCGA and GETx databases and explored their biological roles in glioma. The target protein *MT1-MMP* was screened and the protein interaction network was constructed. The relationship betweenthe expression of *MT1-MMP*, *β1-integrin* and *YAP1* in gliomas and prognosis was verified by GEPIA. Then, we performed an immunohistochemical experiment to detect the expression of three proteins in human gliomas paraffin tissues. The results showed that the expressions of *MT1-MMP*, *β1-integrin* and *YAP1* in GBM were higher than those in LGG. These results provided a preliminary foundation for the later design of several functional tests in this group to determine whether *MT1-MMP* promotes progression of glioma and to evaluate the carcinogenic role of *MT1-MMP* in human cancer by regulating *β1-integrin* in glioma cells.

## 2 Material and methods

### 2.1 Tissue specimens

Paraffin blocks containing representative tumor and hyperplasia samples were selected by reviewing the hematoxylin and eosin-stained slides. The TMA was constructed from a 2 mm diameter core derived from a representative area of a formalin-fixed paraffin-embedded (FFPE) tissue block. Sample size: 214 cases of gliomas, all surgical resections were collected from July 2010 to August 2016 in the First Affiliated Hospital of Xinjiang Medical University. The resected specimens of these 214 patients were diagnosed as glioma in the pathology department based on the latest WHO standards. The WHO II and III gliomas were referred to as LGG (142 cases), whereas the WHO IV gliomas were referred to as GBM (72 cases) [5]. This study was approved by the Ethical Committee of The First Affiliated Hospital of Xinjiang Medical University (Ethics Number: K202103-07), and written informed consent was obtained from all patients.

### 2.2 Reagents and immunohistochemistry

Immunochemistry was performed manually as follows: rabbit monoclonal anti-*MT1-MMP* antibody (ab51074, 1:50, abcam), rabbit monoclonal anti-*β1-integrin* (ab179471, 1:500, abcam) and rabbit monoclonal anti-*YAP1* (ab52771, 1:100, abcam) were purchased for immunohistochemistry test. On the day before the experiment, tissue chips were put into the 37°C oven for the night, softening the wax layer that covered the tissue chip. After dewaxing and dehydration, the tissue chip was put in a boiling EDTA repair solution (pH 8.0) and boiled for 24 min. After 30 min of cooling at room temperature, endogenous peroxidase was added and incubated for 20 min at room temperature. Then, it was rinsed with phosphate-buffered saline 3 times (3 min/time) and added to a 37°C oven for 1 h. Afterward, goat antimouse secondary antibody (PV-6002, Zsbio) was dropped and placed in a 37°C oven for 30 min. Finally, the DAB staining solution was added to obtain a brown color.

### 2.3 Scoring of immunostaining

For each protein, immunohistochemical expression was semi-quantitatively assessed using the immunoreactive score (IRS) as previously described [5], and the cellular localization of immunostaining (membranous, cytoplasmic or nuclear) was also noted. The percentage of positive cells was scored as a four-tiered scale: 0 (staining absent), 1 (≤10%), 2 (10–50%) and 3 (>50%) [5]. The intensity of staining was assessed as follows: 0 (no staining), 1 (weak intensity), 2 (moderate intensity) and 3 (strong intensity). The IRS can range from 0 to 9 and two classes were defined for the study for *MT1-MMP*: negative to weak staining (corresponding to IRS = 0–4) versus moderate to strong staining (corresponding to IRS = 6–9). For *YAP1* and *β1-integrin*: negative to weak staining (corresponding to IRS = 0–3) versus moderate to strong staining (corresponding to IRS = 4–9).

### 2.4 Bioinformatics analysis

Gene Expression Profiling Interactive Analysis (GEPIA; http://gepia.cancer-pku.cn/inde x.html) is a publicly available interactive web server for analyzing gene expression data from cancer and normal tissues from The Cancer Genome Atlas (TCGA) and genotype-tissue expression (GTEx, https://commonfund.nih.gov/gtex) projects. While TCGA only contained five normal brain tissue samples, in this study, we chose the GETx database as the control. The integrated RESM FPKM data and phenotype data (Cohort: TCGA Target GTEX) on UCSC XENA were downloaded to make a heatmap. The UCSC XENA project was used to re-analyze the expression data of TCGA and GTEx with the same standard flow, reducing the batch effect that may have been caused by different data processing methods such as software and parameters as much as possible. FPKM is a gene expression data format that corrects the depth of sequencing. GEPIA was used to analyze the prevalence of a gene signature in TCGA and GTEx samples. The mean value of the log2(TPM + 1) was used as the signature score, and users could calculate the correlation between two signatures and check their prognostic impact [32]. The results of expression of the protein *MT1-MMP*, *β1-integrin*, and *YAP1* and the correlation of three proteins in glioma were further verified by GEPIA to verify the expression of central proteins and OS in this study. The protein–protein interaction (PPI) network relevant to *MT1-MMP* was constructed by the STRINIG database (http://string-db.org/cgi/input.pl). The PPI network was constructed under the condition of: network type: full string network type; the meaning of network edges: evidence; minimum required interaction score: 0.40.

### 2.5 Statistical analysis

Significance was established with Graphpad Prism V.8.0 software and SPSS V.23.0 (IBM). The statistical correlations of immunohistochemical expressions and clinical and pathological features of patients were analyzed using the Pearson’s correlation and *χ* ^2^ test. The relationship between *MT1-MMP*, *β1-integrin* and *YAP1* was determined by Pearson’s correlation and *χ* ^2^ test analysis. Pearson’s correlation was used to determine the association between the expression of *MT1-MMP*, *YAP1*, and *β1-integrin* in LGG with molecular characteristics. PFS was the time interval (months) between the date of surgery and the first tumor progression (or death, or the latest follow-up). OS was defined as the period between the date of initial surgery and the date of the last follow-up or death. Univariate and multivariate analyses were used to determine the survival analysis by the Kaplan–Meier method and Cox-hazard regression analysis. All *p*-values were two-sided, with *p* < 0.05 considered statistically significant.

## 3 Results

### 3.1 Bioinformatics analysis

**Figure 1:**
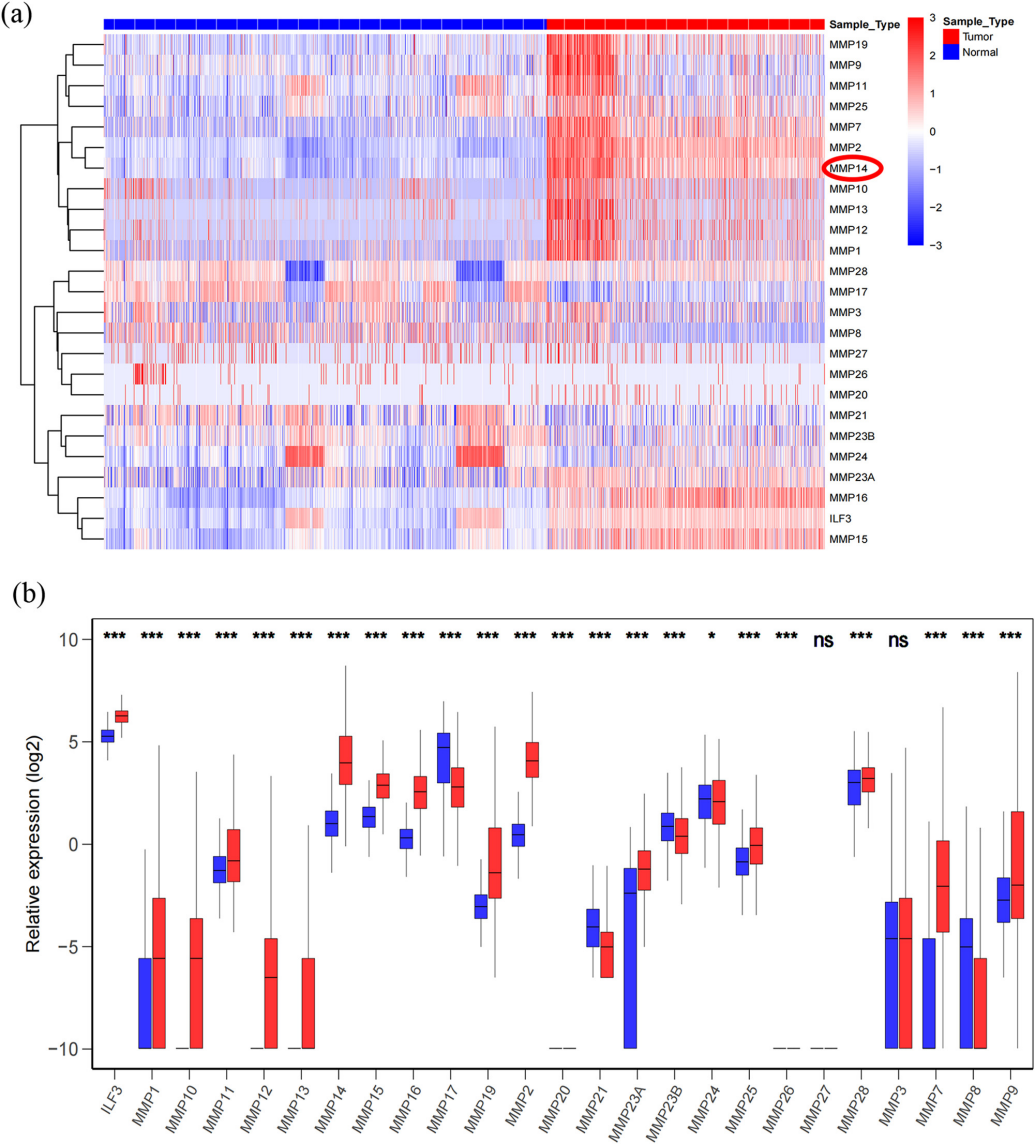
MMP family expression in microarray datasets. (a) Heatmap depicting the MMP expression in microarray datasets (sample: 171 cases GBM + 523 cases LGG + 5 cases normal brain tissue from TCGA database and 1092 cases normal brain tissue from GETx). (b) Relative MMP expression in database. Mann–Whitney *U* test is used to calculate the *p* value. Red indicates high expression; white indicates medium expression; blue indicates lower expression. GBM, glioblastomas; LGG, lower-grade gliomas. ns, not statistically significant. ILF3, MMP4. *p < 0.05, ***p < 0.001.

To investigate the expression of the MMP family in glioma development, we investigated the mRNA expression levels of the nine MMP family members (*MMP1–29*) in The Cancer Genome Atlas (TCGA) and GETx database. The heat map in Figure 1a shows the expression levels of MMP family members in gliomas. The expression of each family member in the glioma tissue and normal brain tissue is shown in Figure 1b. Among these MMP families, *MT1-MMP* was one of the most expressed proteins in gliomas.

**Figure 2:**
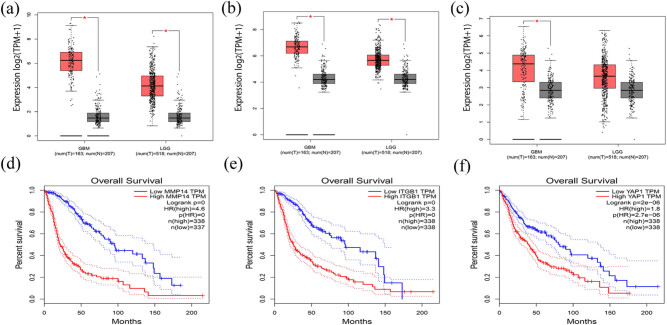
Expression levels and prognostic values of MT1-MMP, β1-integrin and YAP1 in glioma by GEPIA. (a–c) Expression levels of MT1-MMP, β1-integrin and YAP1 in glioma and normal brain tissues. (d–f) Prognostic values of MT1-MMP, β1-integrin and YAP1 in patients with glioma. HR: hazard ratio. GBM: glioblastoma, LGG: lower-grade glioma. *p < 0.05.

**Figure 3:**
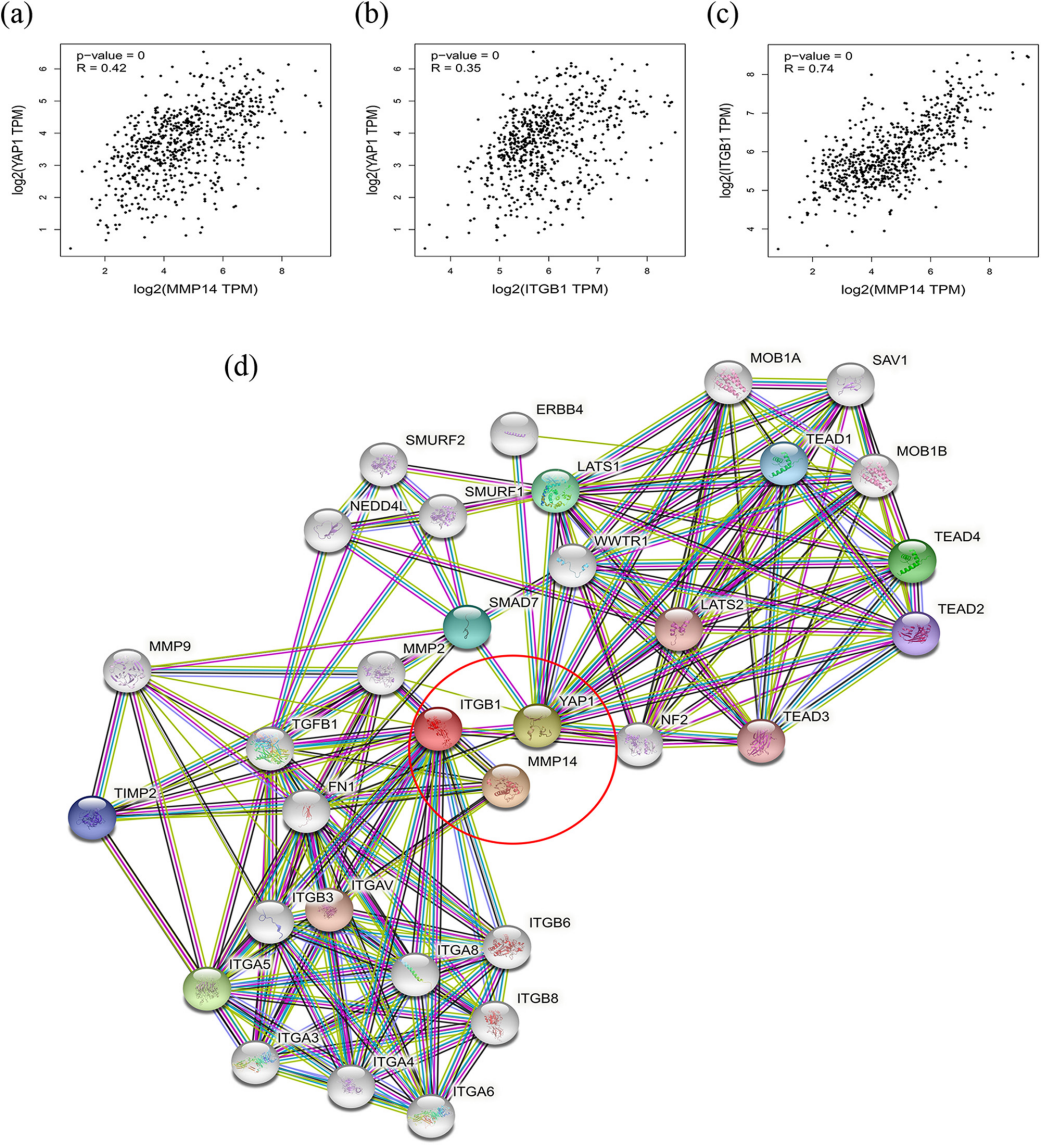
(a) The correlation of MT1-MMP and YAP1 in glioma by GEPIA: a positive correlation between expressions of the two genes (*p* < 0.001, *r* = 0.42). (b) The correlation of β1-integrin and YAP1 in glioma by GEPIA: a positive correlation between expressions of the two genes (*p* < 0.001, *r* = 0.35). (c) The correlation of MT1-MMP and β1-integrin in glioma by GEPIA: a positive correlation between expressions of the two genes (*p* < 0.001, *r* = 0.74). (d) The protein–protein interaction (PPI) network relevant to MT1-MMP (MMP14) was constructed by the STRINIG database (*p* < 0.001).

Next, to ensure the reliability of the identification of the *MT1-MMP*, *β1-integrin*, and *YAP1* genes, we validated these via the GEPIA using TCGA and GTEx databases. Boxplots of the hub genes associated with glioma were downloaded from the GEPIA. The results demonstrated that were significantly overexpressed in GBM and LGG tissues in comparison with normal brain tissues (Figure 2a–c). Patient survival analysis performed via the GEPIA using TCGA and GTEx databases demonstrated that the high expression levels of three proteins were correlated with an unfavorable prognosis in glioma patients (*p* < 0.05) (Figure 2d–f). GEPIA further verified the association of the three proteins in glioma (Figure 3a–c). The PPI network contains 33 genes, with a margin of 181 and a local clustering coefficient of 0.817, indicating significant enrichment of PPI and statistically significant difference (*p* < 0.001) (Figure 3d).

### 3.2 Clinicopathological characteristics

In our study, the clinicopathological characteristics of 214 glioma patients are presented in Table 1. Our cohort consisted of LGG (66.4%) and GBM (33.6%). The numbers of patients diagnosed as each pathological stage were as follows: WHO II 93 (43.5%), WHO III 49 (22.9%), and WHO IV 72 (33.6%). There were 124 men and 90 women, with a median age of 47 ± 14.5 years (range: 5–77), including 105 Han and 109 others; mainly included cases with tumor size > 3 cm (82.7%), origination in frontal lobes (49.5%), tumor total resection (74.8%), no recurrence (58.9%), postoperative radio-chemotherapy (47.7%), Ki-67 expression ≥ 10% (57.9%), and P53 expression ≥ 5% (42.5%). *IDH1* mutation status was available for 142 LGG patients, among whom 117 (83.4%) were wild-type and 25 (17.6%) harbored *IDH1* mutation. Ages (*p* < 0.001), tumor location (*p* < 0.001), selection of the operation method (*p* < 0.001), recur (*p* < 0.001), Ki-67 (*p* < 0.001) and P53 (*p* < 0.001) between patients with LGG and GBM reached statistical significance.

**Table 1:** Clinicopathological characteristics of all glioma patients

Clinicopathologic characteristics	Total (214)	LGG (II + III, *n* = 142)	GBM ( *n* = 72)	*p*-value
Gender				
Male	124(124/214,57.9%)	82(82/124,38.3%)	42(42/124,19.6%)	0.527
Female	90(90/214,42.1%)	60(60/90,28.0%)	30(30/90,14.0%)	
Age				
<50	120(120/214,56.1%)	99(99/120,46.3%)	21(42/120,9.8%)	<0.001*
≥50	94(94/214,43.9%)	43(43/94,20.1%)	51(51/94,23.8%)	
Ethnic				
Han	105(105/214,49.1%)	73(73/105,34.1%)	32 (32/105,15.0%)	0.207
Other	109(109/214,50.9%)	69(69/109,32.2%)	40(40/109,18.7%)	
Tumor location				
Frontal	106(106/214,49.5%)	57(57/106,26.6%)	49(49/106,22.9%)	<0.001*
Other	108(108/214,50.5%)	85(85/108,39.7%)	23(23/108,10.7%)	
Tumor size				
<3 cm	37(37/214,17.3%)	25(25/37,11.7%)	12(12/37,5.6%)	0.513
≥3 cm	177(177/214,82.7%)	117(117/177,54.7%)	60(60/177,28.0%)	
Selection of operation method				
Total resect	160(160/214,74.8%)	117(117/160,54.7%)	43(43/160,20.1%)	<0.001*
Partial resect	54(54/214,25.2%)	25 (25/54,11.7%)	29(29/54,13.6%)	
Postoperative radiochemotherapy				
Yes	102(102/214,47.7%)	63(63/102,29.4%)	39(39/102,18.2%)	0.113
No	112(112/214,52.3%)	79(79/112,36.9%)	33(33/112,15.4%)	
Recur				
Yes	88(88/214,41.1%)	19(19/88,8.9%)	69(69/88,32.2%)	<0.001*
No	126(126/214,58.9%)	123(123/126,57.5%)	3(3/126,1.4%)	
Ki-67				
<10%	90(90/214,42.1%)	84(84/90,39.3%)	6(6/90,2.8%)	<0.001*
≥10%	124(124/214,57.9%)	58(58/124,27.1%)	66(66/124,30.8%)	
P53				
<5%	123(123/214,57.5%)	95(95/123,44.4%)	28(28/123,13.1%)	<0.001*
≥5%	91(91/214,42.5%)	47(47/91,22.0%)	44(44/91,20.6%)	

Statistical analyses were performed by the Pearson *χ* ^2^ test. *p < 0.05 was considered statistically significant. GBM, glioblastomas; LGG, lower-grade gliomas.

### 3.3 Association between the expression levels of MT1-MMP, β1-integrin, YAP1 and clinicopathological characteristics

**Figure 4:**
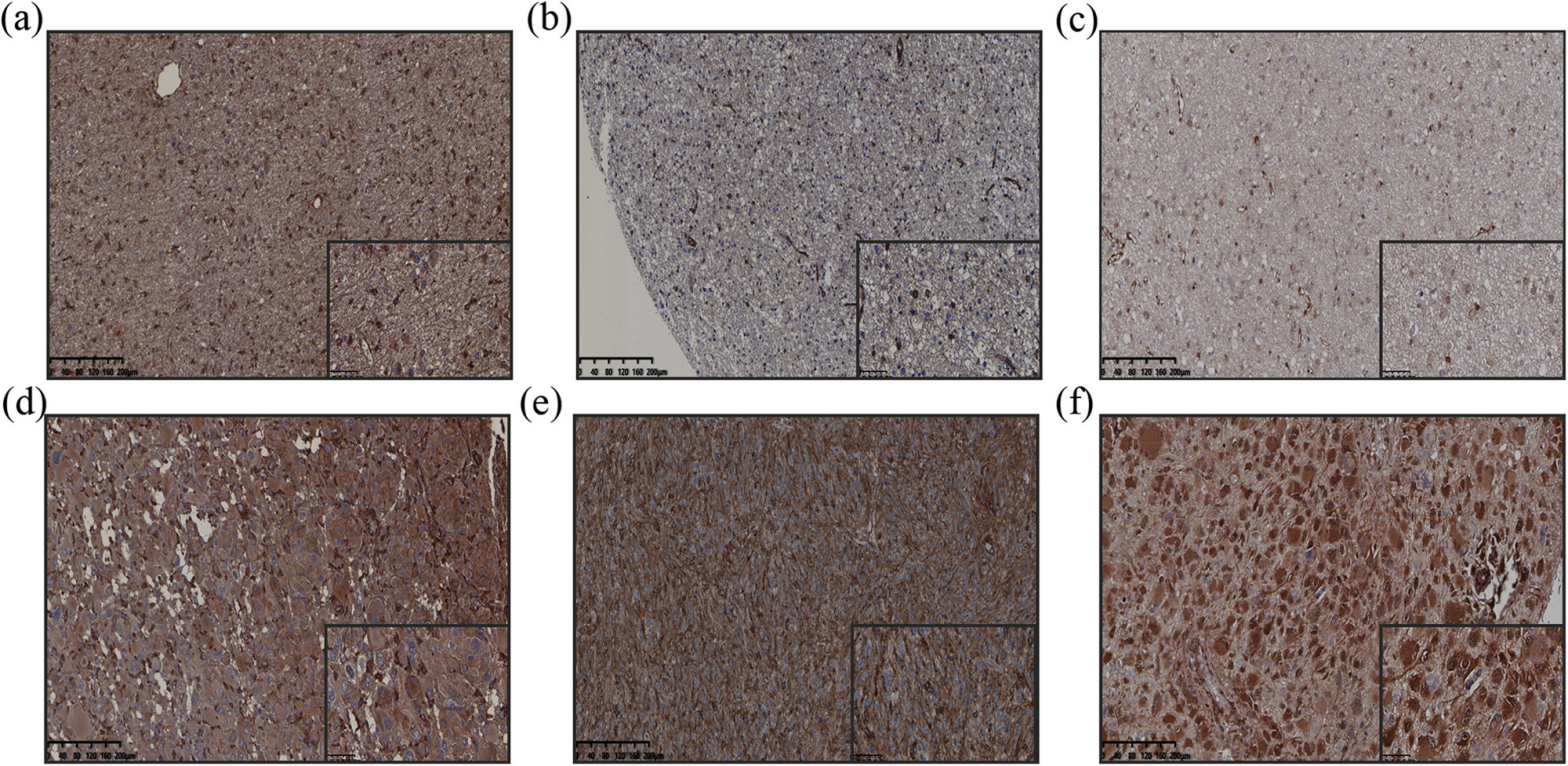
Immunohistochemistry analysis of MT1-MMP, β1-integrin and YAP1 expression in glioma tissues. (a) MT1-MMP expression in LGG (200×). (b) β1-integrin expression in LGG (200×). (c) YAP1 expression in LGG (200×). (d) MT1-MMP expression in GBM (200×). (e) β1-integrin expression in GBM (200×). (f) YAP1 expression in GBM (200×). GBM, glioblastomas; LGG, lower-grade gliomas.

**Figure 5:**
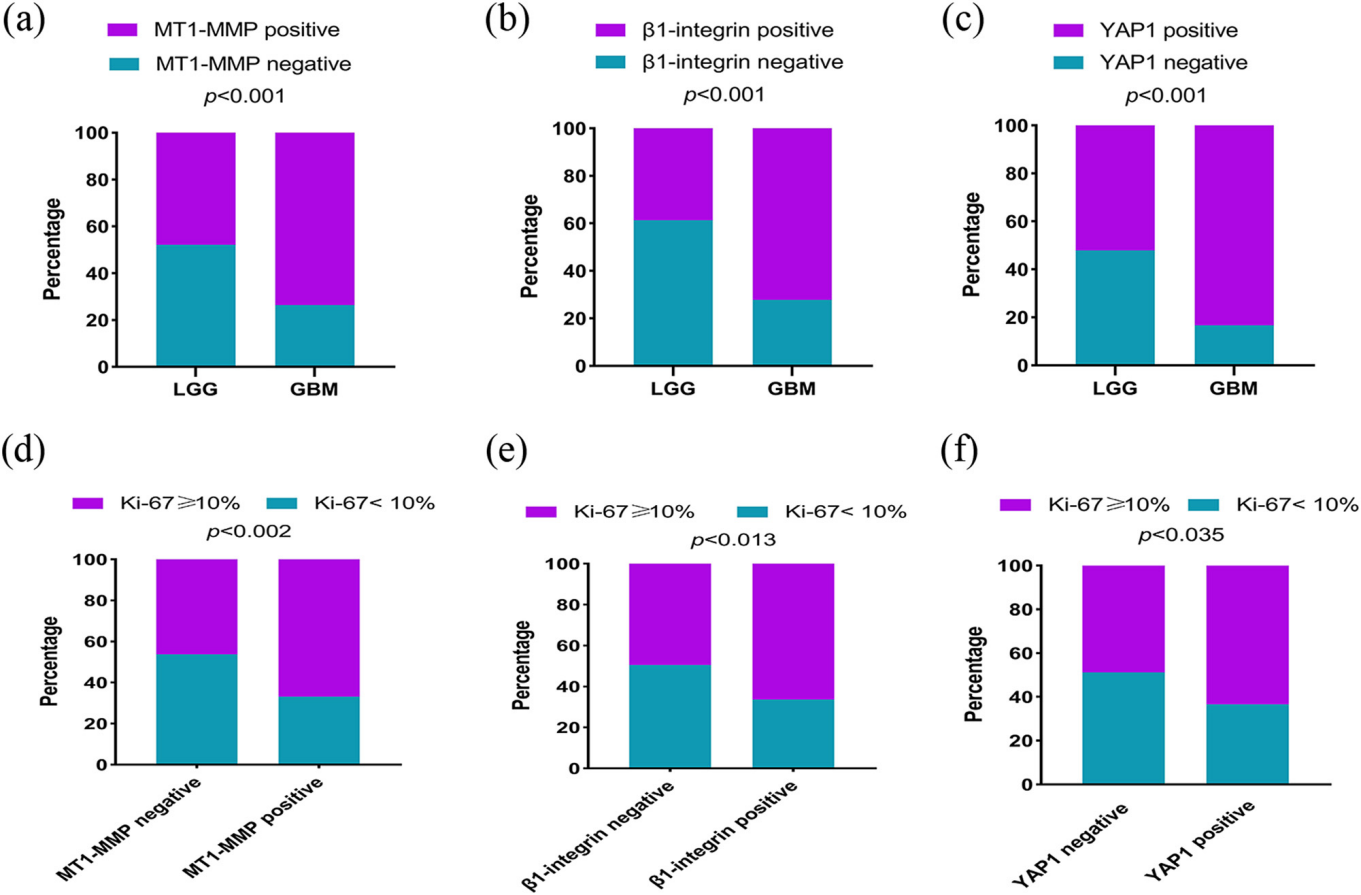
The expression levels of MT1-MMP, β1-integrin, YAP1 and Ki-67 in glioma. (a) MT1-MMP high expression in 47.9% LGG and 73.6% GBM. (b) β1-integrin high expression in 38.7% LGG and 72.2% GBM. (c) YAP1 high expression in 52.1% LGG and 83.3% GBM. (d) Ki-67 high expression in 20.1% MT1-MMP negative and 37.9% MT1-MMP positive. (e) Ki-67 high expression in 18.2% β1-integrin negative and 39.7% β1-integrin positive. (f) Ki-67 high expression in 24.8% YAP1 negative and 33.2% YAP1 positive. GBM, glioblastomas; LGG, lower-grade gliomas.

The expression of *MT1-MMP* is localized on the membrane *and* cytoplasm of tumor cells. *β1-Integrin* is normally expressed in the cell cytoplasm. As *YAP1* is a transcriptional coactivator that shuttles between the cytoplasm and nucleus, we evaluated both its cytoplasmic and nuclear expression in the human glioma specimens (Figure 4a–f). In all cohorts (LGG and GBM), there were 121 (56.5%) positive cases and 93 (56.5%) negative cases of *MT1-MMP*. Further, in 72 GBM and 142 LGG (II and III), we found that the cases with *MT1-MMP* positive expression only accounted for 68 (47.9%) in LGG and 53 (73.6%) in GBM (Figure 5a). The high expression of *MT1-MMP* was prominent in GBM, increasing the aggressiveness of the tumor. Statistical analysis revealed that *MT1-MMP* expression was related to age (*p* = 0.003), WHO grade (*p* = 0.001), recurring (*p* = 0.010) and P53 expression (*p* = 0.035). There were 107 (50.0%) cases of positive expression of *β1-integrin* and 107 (50.0%) cases of negative. Further, we found that the cases with *β1-integrin* positive expression only accounted for 55 (38.7%) in LGG and 52 (72.2%) in GBM (Figure 5b). The results revealed that *β1-integrin* expression was related to WHO grade (*p* = 0.001) and recurring (*p* = 0.001) and not to P53 expression (*p* = 0.128). *YAP1* was positive in 134 cases and negative in 80 cases of glioma. Further, we found that the cases with *YAP1* positive expression only accounted for 74 (52.1%) in LGG and 60 (83.3%) in GBM (Figure 5c). Statistical analysis revealed that *YAP1* expression was related to age (*p* = 0.009), ethnic (*p* = 0.029), WHO grade (*p* = 0.001), recurring (*p* = 0.004) and P53 expression (*p* = 0.001) in glioma (Tables 2 and 3). The association between the positive expression of the three proteins and the clinicopathological parameters was assessed in 214 patients with glioma. Compared with LGG, the expression levels of *MT1-MMP*, *β1-integrin* and *YAP1* were higher in the tissues of patients with GBM.

**Table 2:** Correlation between MT1-MMP, β1-integrin, YAP1 expression and clinicopathological parameters in glioma

Clinicopathologic characteristics	MT1-MMP			β1-integrin			YAP1		
	Negative	Positive		Negative	Positive		Negative	Positive	
Gender									
Male	54(25.2%)	70(32.7%)	0.975	60(28.0%)	64(29.9%)	0.580	43(20.1%)	81(37.9%)	0.337
Female	39(18.2%)	51(23.8%)		47(22.0%)	43(29.9%)		37(17.3%)	53(24.8%)	
Age									
<50	63(29.4%)	57(26.6%)	0.003*	65(30.4%)	55(25.7%)	0.168	54(25.2%)	66(30.8%)	0.009*
≥50	30(14.0%)	64(29.9%)		42(19.6%)	52(24.3%)		26(12.1%)	68(31.8%)	
Ethnic									
Han	51(23.8%)	54(25.2%)	0.139	52(24.3%)	53(24.8%)	0.891	47(22.0%)	58(27.1%)	0.029*
Other	42(19.6%)	67(31.3%)		55(25.7%)	54(25.2%)		33(15.4%)	76 (35.5%)	
Tumor location									
Frontal	44(20.6%)	62(29.0%)	0.569	50(23.4%)	56(26.2%)	0.412	37(17.3%)	69(32.2%)	0.458
Other	49(22.9%)	59(27.6%)		57(26.6%)	51(23.8%)		43 (20.1%)	65(30.4%)	
Tumor size									
<3 cm	13(6.1%)	24(11.2%)	0.261	20(9.3%)	17(7.9%)	0.588	14(6.5%)	23(10.7%)	0.950
≥3 cm	80(37.4%)	97(45.3%)		87(40.7%)	90(42.1%)		79 (36.9%)	98 (45.8%)	
WHO grade									
WHO II	52(24.3%)	41(19.2%)	0.001*	61(28.5%)	32(15.0%)	0.001*	43(20.1%)	50(23.4%)	0.001*
WHO III	22(10.3%)	27(12.6%)		26(12.1%)	23(10.7%)		25(11.7%)	24(11.2%)	
WHO IV	19(8.9%)	53(24.8%)		20(9.3%)	52(24.3%)		12(5.6%)	60(28.0%)	
Selection of operation method									
Total resect	74(34.6%)	86(40.2%)	0.156	86(40.2%)	74(34.6%)	0.393	63(29.4%)	97(45.3%)	0.300
Partial resect	19(8.9%)	35(16.4%)		21(9.8%)	33(15.4%)		17(7.9%)	37(17.3%)	
Postoperative radiochemotherapy									
Yes	44(20.6%)	58(27.1%)	0.928	48(22.4%)	54(25.2%)	0.412	38(17.8%)	64(29.9%)	0.970
No	49(22.9%)	63(29.4%)		59(27.6%)	53(24.8%)		42(19.6%)	70(32.7%)	
Recur									
Yes	29(13.6%)	59(27.6%)	0.010*	79(36.9%)	47(22.0%)	0.001*	23(10.7%)	65(30.4%)	0.004*
No	64(29.9%)	62(29.0%)		28(13.1%)	60(28.0%)		57(26.6%)	69(32.2%)	
P53									
<5%	61(28.5%)	62(29.0%)	0.035*	67(31.3%)	56(26.2%)	0.128	59(27.6%)	64(29.9%)	0.001*
≥5%	32(15.0%)	59(27.6%)		40(18.7%)	51(23.8%)		21(9.8%)	70(32.7%)	

Statistical analyses were performed by the Pearson *χ* ^2^ test. * *p* < 0.05 was considered statistically significant.

**Table 3:** Staining results of different antibodies on the glioma specimens (*n* = 214)

Proteins	Grade	Negative	Positive	*p*-value
MT1-MMP	LGG	74(74/142,52.1%)	68(68/142,47.9%)	0.001*
	GBM	19(19/72,26.4%)	53(53/72,73.6%)	
β1-Integrin	LGG	87(87/142,61.3%)	55(55/142,38.7%)	0.001*
	GBM	20(20/72,27.8%)	52(52/72,72.2%)	
YAP1	LGG	68(68/142,47.9%)	74(74/142,52.1%)	0.001*
	GBM	12(12/72,16.7%)	60(60/72,83.3%)	

*p* values were calculated by Pearson’s *χ* ^2^ test (two sided). *p < 0.05 indicates statistical significance. GBM, glioblastomas; LGG, lower-grade gliomas.

In order to further explain the role of *MT1-MMP*, *β1-integrin*, *YAP1* in glioma, we examined the relationship between three proteins and Ki-67 (Table 4) (Figure 5d–f). The final results showed that there was statistical significance for the correlation between *MT1-MMP* (*r* = 0.208, *p* = 0.002), *YAP1* (*r* = 0.144, *p* = 0.035), *β1-integrin* (*r* = 0.170, *p* = 0.013) with Ki-67.

**Table 4:** Correlation between MT1-MMP, YAP1, β1-integrin and Ki-67

Proteins	Expression	Ki-67		*r*	*p*-value
		+	−		
MT1-MMP	+	81(37.9%)	40(18.7%)	0.208	0.002*
	−	43(20.1%)	50(23.4%)		
YAP1	+	85(39.7%)	49(22.9%)	0.144	0.035*
	−	39(18.2%)	41(19.2%)		
β1-Integrin	+	71(33.2%)	36(16.8%)	0.170	0.013*
	−	53(24.8%)	54(25.2%)		

*p < 0.05 indicates statistical significance.

### 3.4 The relationship between MT1-MMP, β1-integrin and YAP1 in glioma

In 214 cases of glioma, Pearson’s correlation analysis was performed to clarify the associations among *MT1-MMP*, *β1-integrin* and *YAP1*. The result showed that the expression of *YAP1* was positively correlated with *MT1-MMP* (*p* = 0.001, *r* = 0.443) and *β1-integrin* (*p* = 0.001, *r* = 0.348). At the same time, the expression of *MT1-MMP* was correlated with *β1-integrin* (*p* = 0.001, *r* = 0.387) (Table 5).

**Table 5:** Correlation between MT1-MMP, YAP1 and β1-integrin

Protein	Expression	+	−	*r*	*p*-value
**YAP1**					
MT1-MMP	+	98(45.8%)	23(10.7%)	0.443	<0.001*
	−	36(16.8%)	57(26.6%)		
β1-Integrin	+	85(39.7%)	22(10.3%)	0.348	<0.001*
	−	49(22.9%)	58(27.1%)		
**MT1-MMP**					
β1-Integrin	+	81(37.9%)	26(12.1%)	0.387	<0.001*
	−	40(18.7%)	67(31.3%)		

^*^ *p* < 0.05 indicates statistical significance.

### 3.5 The expression of MT1-MMP, YAP1, and β1-integrin in LGG with molecular characteristics

**Figure 6:**
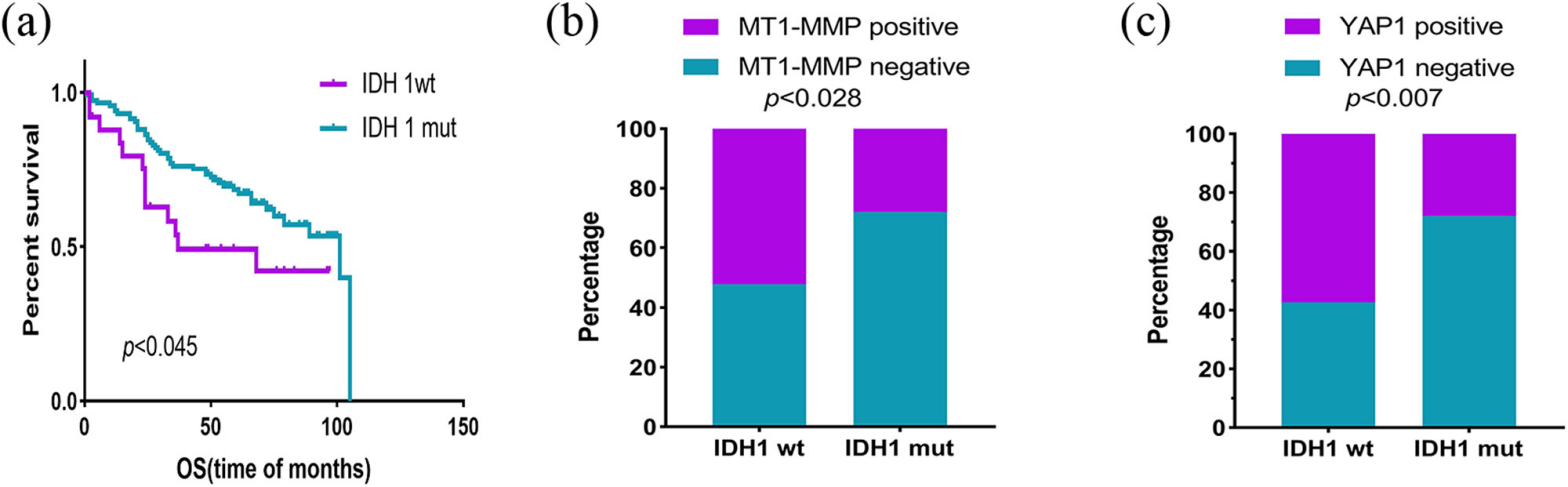
Expression of IDH1 in LGG and their relationship with prognosis. (a) Patients with IDH1 mutation had a longer OS time. (b) MT1-MMP was expressed significantly differently between IDH1mut and IDH1wt. (c) YAP1 was expressed differently between IDH1mut and IDH1wt. IDH1mut, IDH1 mutation; IDH1wt, IDH1wild type; LGG, lower-grade gliomas; OS, overall survival.

For 142 LGG, there were 117 cases of *IDH1* mutantion (*IDH1* mut) and 25 cases of *IDH1* wild type (*IDH1* wt), including 62 cases 1p19q codeletion and 80 cases non-codeletion. Next, we examined the relationship among *MT1-MMP, YAP1* and *β1-integrin* with molecular typing (Table 6). *IDH1* wt showed worse OS of glioma patients (*p* = 0.045) (Figure 6a). The results showed that the expression of *MT1-MMP* (*r* = 0.260, *p* = 0.002) and *YAP1* (*r* = 0.221, *p* = 0.008) were correlated with *IDH1* statistically (Figure 6b and c).

**Table 6:** The expression of MT1-MMP, β1-integrin and YAP1 in LGG according to molecular characteristics

		IDH 1		*r*	*p*-value	1p19q		*r*	*p*-value
Proteins		mut	wt			non-codel	codel		
MT1-MMP	Negative	68(68/117,47.9%)	6(6/25,4.2%)	0.260	0.002*	42(42/80,29.6%)	32(32/62,22.5%)	0.009	0.917
	Positive	49(49/117,34.5%)	19(19/25,13.4%)			38(38/80,26.8%)	30(30/62,21.1%)		
YAP1	Negative	62(62/117,43.7%)	6(6/25,4.2%)	0.221	0.008*	36(36/80,25.4%)	32(32/62,22.5%)	−0.066	0.438
	Positive	55(55/117,38.7%)	19(19/25,13.4%)			44(44/80,31.0%)	30(30/62,21.1%)		
β1-integrin	Negative	74(74/117,52.1%)	13(13/25,9.2%)	0.088	0.298	47(47/80,33.1%)	40(40/62,28.2%)	−0.059	0.488
	Positive	43(43/117,30.3%)	10(10/25,8.5%)			33(33/80,23.2%)	22(22/62,15.5%)		

*p < 0.05 indicates statistical significance.

### 3.6 Prognostic significance: univariate and multivariate analyses of OS and PFS

**Figure 7:**
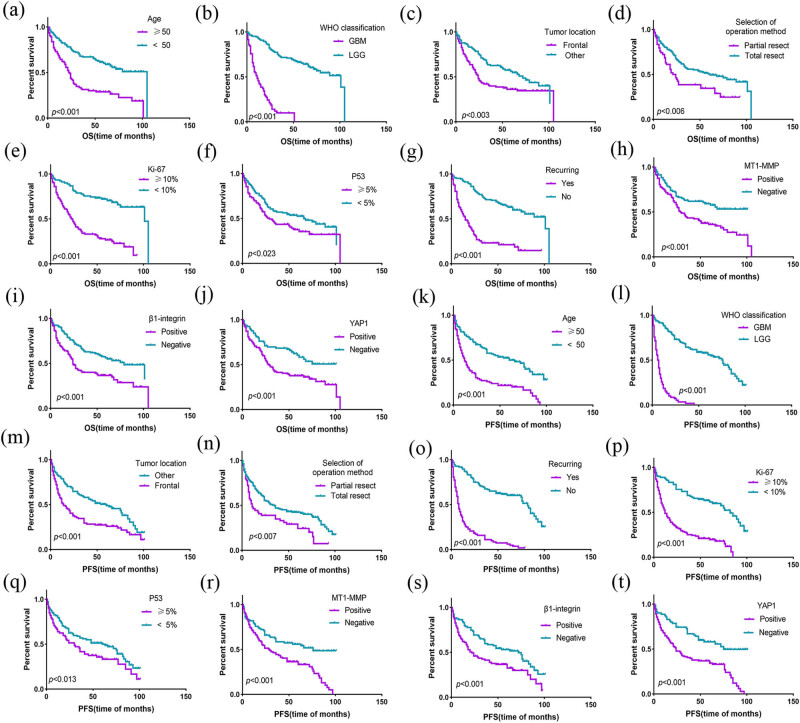
Kaplan–Meier survival analysis for overall survival and progression-free survival. (a–j) Results of univariate survival analysis by the Kaplan–Meier method on OS (*p* < 0.05). (k–t) Results of univariate survival analysis by the Kaplan–Meier method on PFS (*p* < 0.05).

To assess the prognostic significance of the expression of three proteins and the clinical characteristics in glioma patients, we constructed univariate and multivariate analyses of OS and PFS. Survival analysis was conducted in the total cohort (Table 7). For 214 cases of glioma, Univariate analysis by the Kaplan–Meier survival analysis showed that the following prognostic factors exhibited an effect on OS (Figure 7): age (*χ* ^2^ = 28.456, *p* < 0.001), tumor location (*χ* ^2^ = 9.051, *p* = 0.003), WHO classification (*χ* ^2^ = 129.325, *p* < 0.001), selection of operation method (*χ* ^2^ = 7.418, *p* = 0.006), recurring (*χ* ^2^ = 59.024, *p* < 0.001), Ki-67 (*χ* ^2^ = 46.002, *p* < 0.001), p53 (*χ* ^2^ = 5.177, *p* = 0.023), *MT1-MMP* (*χ* ^2^ = 11.647, *p* = 0.001), *β1-integrin* (*χ* ^2^ = 11.392, *p* = 0.001), and *YAP1* (*χ* ^2^ = 12.464, *p* = 0.001). Multivariate analysis (Cox’s proportional hazards regression model) on OS showed that the possible independent prognostic factors for OS were as follows: age (HR: 1.479; 95 % CI: 0.999–2.189; *p* = 0.050), WHO classification (HR: 4.978; 95 % CI: 3.091–8.017; *p* < 0.001), and Ki-67 (HR: 2.241; 95 % CI: 1.371–3.665; *p* = 0.001). In univariate survival analysis of PFS, the prognostic factors were the same as those of OS, and in multivariate analysis of PFS, WHO classification (HR: 3.392; 95 % CI: 1.01–6.388; *p* < 0.001), recurring (HR: 2.217; 95 % CI: 1.227–4.009; *p* = 0.008), Ki-67 (HR: 1.875; 95 % CI: 1.217–2.889; *p* = 0.004) and *YAP1* expression (HR: 1.634; 95 % CI: 1.113–2.397; *p* = 0.012) were independent prognostic factor for PFS.

**Table 7:** Univariable and multivariable analysis for overall survival and progression-free survival

Variable	Overall survival				Progression-free survival			
	Univariable		Multivariable		Univariable		Multivariable	
	*χ* ^2^	*p*-value	HR (95% CI)	*p*-value	*χ* ^2^	*p*-value	HR (95% CI)	*p*-value
Gender								
(Female vs male)	2.219	0.136	—	—	0.385	0.535	—	—
Age								
(<50 vs ≥50)	28.456	<0.001*	1.479(0.999–2.189)	0.050*	32.467	<0.001*	—	—
Ethnic								
(Han vs other)	0.089	0.765	—	—	0.084	0.772	—	—
Tumor location								
(Frontal vs other)	9.051	0.003*	—	—	13.980	<0.001*	—	—
Tumor size								
(<3 cm vs ≥3 cm)	0.174	0.677	—	—	0.000	0.998	—	—
WHO classification								
(II & III vs IV)	129.325	<0.001*	4.978(3.091–8.017)	<0.001*	169.283	<0.001*	3.392(1.01–6.388)	<0.001*
Selection of operation method								
(Total resect vs partial resect)	7.418	0.006*	—	—	7.291	0.007*	—	—
Postoperative radiochemotherapy								
(Yes vs no)	0.005	0.946			2.207	0.137	—	—
Recur								
(Yes vs no)	59.024	<0.001*	—	—	112.214	<0.001*	2.217(1.227–4.009)	0.008*
Ki-67								
(<10% vs ≥10%)	46.002	<0.001*	2.068(1.275–3.354)	0.003*	51.104	<0.001*	1.875(1.217–2.889)	0.004*
P53								
(<5% vs ≥5%)	5.177	0.023*	—	—	6.207	0.013*	—	—
MT1-MMP expression								
(Positive vs negative)	11.647	0.001*	—	—	14.801	<0.001*	—	—
β1-Integrin expression								
(Positive vs negative)	11.392	0.001*	—	—	10.845	0.001*	—	—
YAP1 expression								
(Positive vs negative)	12.464	0.001*	—	—	19.979	<0.001*	1.634(1.113–2.397)	0.012*

Survival analysis was conducted by univariate survival analysis (Kaplan–Meier method) and multivariate analysis (Cox regression analysis model). *p < 0.05 indicates statistical significance.

## 4 Discussion

Regardless of grade, gliomas oppress the normal brain tissue during growth, with high mortality, high morbidity and poor prognosis, especially in the most aggressive glioblastoma. The change in the expression levels of certain factors is the characteristic feature of glioma and the prerequisite for tumor development. Therefore, it is very important to clarify the molecular mechanism of glioma progression and screen molecular markers to prevent malignant progression. In this study, TCGA and GETx studied LGG and GBM. We screened the MMPs family in the TCGA and GETx databases and found that *MT1-MMP* was one of the most highly expressed MMPs in gliomas. According to Tang et al. [29], *MT1-MMP* could activate *β1-integrin* to promote the entry of *YAP* and *TAZ* into the nucleus, thus affecting the SSC lineage commitment.

Many studies have shown that *MT1-MMP* plays an important role in angiogenesis, EMT and tumor immunity [33,34,35]. Therefore, defining the role of *MT1-MMP* in tumor development remains a key interest in targeted chemotherapy resistance therapy. *MT1-MMP* can be used as the main vector of matrix remodeling in malignant tumor invasion, tumor metastasis and poor prognosis. Increased mRNA and protein levels of MT1-MMP are detected in nasopharyngeal carcinoma, which regulates the expression of genes related to migration, invasion and EMT of nasopharyngeal carcinoma cells [36]. Through the transwell co-culture system, it was found that astrocytes could induce MT1-MMP activity and enhance the migration and invasion of tumor cells [37], and Chen et al. carried out immunohistochemical experiments on human glioma specimens. The results showed that the group with high expression of MT1-MMP was positively correlated with advanced WHO grade (*p* < 0.001) and low survival rate (*p* < 0.001) [37]. Our present study was consistent with the results reported in previous studies.

*β1-Integrin* expression is highly upregulated in glioblastoma and has been shown to drive tumor progression, invasion, and drug resistance to multiple therapies [38]. *MT1-MMP* is located in the adhesion complex containing *β1-integrin* [39]. *MT1-MMP* is the upstream regulator of *β1-integrin*. Activating *β1-integrin* can promote the invasive sprouting phenotype of melanoma cells [40]. *MT1-MMP* has been shown to activate *β1-integrin* through collagen processing [29]. Charles et al. found that *MT1-MMP* can activate *β1-integrin* to increase the resistance of melanoma to BRAFi by co-transducing the parents and resistant cells expressing *shMT1-MMP* with the construction of self-clustering *β1-integrin* mutant [41]. Grafinger found that activation of *β1-integrin* could induce the phosphorylation and internalization of *MT1-MMP* and increase the expression level of *MT1-MMP*, leading to the invasiveness and the formation of invadopium *in vitro* [42]. In the present study, the *β1-integrin* overexpression group exhibited a poor prognosis based on the OS ratios. However, multivariate survival analyses for *β1-integrin* and poor prognosis exhibited no statistically significant difference. The difference between the aforementioned results may be associated with the individual differences and sample size of the subjects. Moreover, our result demonstrated a significant correlation between the expression of *MT1-MMP* and *β1-integrin* (*p* = 0.001). This is the first time that there is a statistical correlation between *MT1-MMP* and *β1-integrin* in gliomas. Although it provides limited significance, it provides a reference value for the diagnosis and prognosis of gliomas to some extent.

Hippo signaling plays a significant role in the progression of some glioma cancers and their prognosis to chemotherapy [5,43]. *YAP1* is the core effect factor of this pathway and the focus of tumor development at present. We found that high expression of *YAP1* was negatively correlated with the WHO grade and survival rate, indicating that *YAP1* may contribute to tumor proliferation and invasion. The nuclear localization of *YAP* is affected by *MT1-MMP* [29]. On firm substrates, *YAP/TAZ* enters the nucleus and binds to the *PROX-1* promoter to inhibit transcription of *PROX-1* and its targets *MT1-MMP* [44,45]. Conversely, cytoplasmic degradation of *YAP/TAZ* enhances the transcription of *PROX-1*, including its targets *MT1-MMP* [46]. In addition, Christopher et al. found that *YAP* can regulate direct matrix remodeling by osteocytes, and conditional *YAP* deletion can lead to decreased mRNA expression of *MT1-MMP* [47]. This shows that *MT1-MMP* and *YAP1* may regulate and influence each other. In this study, our results only showed that there was a significant correlation between *MT1-MMP* and *YAP1*. To the best of our knowledge, the correlation between *MT1-MMP* and *YAP1* has not been reported previously and the results shown here warrant future investigation. The deficiency was that no more functional experiments had been carried out to further explore the regulation mechanism between them. Although this conclusion is superficial, it lays a theoretical foundation for our later functional experiments.

Studies have shown that silencing *β1-integrin* can suppress the expression and intracellular translocation of *YAP1*, a downstream effector of *β1-integrin*, and induce radioresistance via *YAP1*-induced EMT of NSCLC cells [48]. Yamashiro et al. found that knockdown of *β1-integrin* could inhibit nuclear shuttling of YAP [49]. As we have discussed above, several studies have demonstrated the correlation between *MT1-MMP*, *β1-integrin* and *YAP1*, respectively, but there are a few studies on the three proteins in gliomas. Our study demonstrated a significant correlation between the three proteins in gliomas for the first time but only at the histological level. Whether this conclusion cannot infer that there is a carcinogenic signal pathway of *MT1-MMP/β1-integrin/YAP1* in gliomas needs further study. We are planning to conduct experiments in the later stage to further confirm this. Additionally, our results showed that Ki-67, as a marker of cell proliferation, had a positive correlation with three proteins. It was suggested that *MT1-MMP*, *β1-integrin* and *YAP1* might promote the proliferation of glioma cells and eventually lead to the malignant progression of gliomas.

Glioma cells, but not microglia cells, can promote cytoplasmic matrix remodeling by producing *MT1-MMP*, thereby promoting tumor tissue infiltration into the surrounding brain tissue, and in diffuse gliomas, the majority of *MT-MMP*-producing (including *MT1-MMP*) cells were *IDH*-mutated tumor cells. In other words, tumor cells are the main source of *MT1-MMP* in gliomas with diffuse *IDH* mutations, which provides a basic theoretical basis for this study to analyze the relationship between *MT1-MMP* and *IDH1* at the histological level [50]. However, other studies have shown that Iba1 immunoreactive microglia can express 28–91% of *MT1-MMP* in diffuse gliomas and glioblastomas [51]. Our results showed a significant correlation between *MT1-MMP* and *IDH1*, consistent with that reported by Thome et al. [50]. Whether glioma cells or microglia produce *MT1-MMP* needs further study in this group. Guichet et al. found that the expression level of *YAP1* in *IDH*wt tumors was higher than that in *IDH*mut tumors, showing a lower level of methylation. This result could explain the expression of *YAP1* between *IDH*mut and *IDH*wt [5]. Shuang et al. found that *YAP1* was a new molecular target of *IDH1* ^*R132H/WT*^ by using the “single base editing” technique in human diffusion astroglioma. It suggested that *YAP1* was downregulated by about 50% in *IDH1* ^*R132H/WT*^ cells, but the methylation level of *YAP1* had not been changed. They also identified the signal transduction network of the *YAP1* pathway in *IDH1* ^*R132H/WT*^ cells, and the results showed that *YAP1* mediated the antiproliferation effect of *IDH1* ^*R132H/WT*^ through *NOTCH* [52]. Our results showed a statistically significant correlation between *YAP1* and *IDH1*. This result was consistent with previous studies [5,53]. So far, no research had found any relationship between *β1-integrin* and *IDH1*, and our study also showed that there was no significant correlation between them. This needs further research. In summary, this suggested that *MT1-MMP* and *YAP1* may be a prognostic factor and therapeutic target by affecting the molecular typing of glioma. However, our results do not account for the statistical relationship between three proteins and 1p19q. *MT1-MMP* and *YAP1* may affect the *IDH1* molecular typing of LGG, although further experimental verification was needed.

These results showed that the independent factor that can predict short OS and PFS in the total glioma cohort was WHO grade. Besides, age was the independent prognostic factor for OS. The expression level of *YAP1* and recurring could also predict the PFS in glioma. These data provided rich evidence that *YAP1* could be used as an independent prognostic and diagnostic marker for glioma and could be used to assess the survival of patients with glioma. It was more conducive to accurately predict the prognosis and survival time of patients and the conclusions were more comprehensive and specific among different clinicopathological parameters. By comparison, we can predict the prognosis and survival of glioma patients with different clinicopathological parameters. And, in the future, there will be more research on the carcinogenic role of *YAP1* in glioma, which is expected to achieve targeted personalized treatment of glioma patients with *YAP1*.

In summary, our analysis not only broadens understanding of the carcinogenic roles of *MT1-MMP*, *β1-integrin* and *YAP1*. They may be involved in the occurrence and development of glioma through related metabolism, which has a certain reference value for judging the prognosis of glioma. *MT1-MMP*, *β1-integrin* and *YAP1* may be effective molecular markers for diagnosing and predicting tumor development. Whether *YAP1* can be used as targets for the treatment of glioma cancer remains to be further studied in this group.
